# PAX3 Expression in Normal Skin Melanocytes and Melanocytic Lesions (Naevi and Melanomas)

**DOI:** 10.1371/journal.pone.0009977

**Published:** 2010-04-22

**Authors:** Sandra Medic, Mel Ziman

**Affiliations:** 1 School of Exercise, Biomedical and Health Sciences, Edith Cowan University, Perth, Western Australia, Australia; 2 School of Pathology and Laboratory Medicine, University of Western Australia, Perth, Western Australia, Australia; The University of Queensland, Australia

## Abstract

**Background:**

Cutaneous Malignant Melanoma is an aggressive form of skin cancer, arising in cutaneous melanocytes. The transcription factor PAX3 regulates melanocyte specification from neural crest cells during development but expression in differentiated melanocytes is uncertain. By contrast it is frequently found in melanomas and naevi and is a marker for melanoma staging and detection. In this study we analysed the expression of PAX3 across the spectrum of melanocytic cells, from normal melanocytes to cells of benign and malignant lesions to better assess its function in these various tissues. *Pax3* and *PAX3* (italicized) refer to the mouse and human gene, respectively; whereas Pax3 and PAX3 (non-italicized) refer to the corresponding mouse and human protein.

**Methodology and Principal Findings:**

PAX3 expression was analysed by immunohistochemistry and qRT-PCR. Immunofluorescence was used for co-expression with differentiation, migration and survival markers. As expected PAX3 expression was observed in naevi and melanoma cells. It was also found in melanocytes of normal skin where it co-expressed with melanocyte markers, MITF and MLANA. Co-expression with its downstream target, antiapoptotic factor BCL2L1 confirms PAX3 as a cell survival regulator. PAX3 was also co-expressed with melanoma cell migration marker MCAM in dermal naevi and melanoma cell nests, but this downstream target of PAX3 was not present in normal epidermal melanocytes, suggesting differential roles for PAX3 in normal epidermal melanocytes and melanoma cells. Most interestingly, a proportion of PAX3-positive epidermal melanocytes in normal skin show HES1 and Ki67 co-expression, indicating their less differentiated proliferative phenotype.

**Conclusions and Significance:**

Our results suggest that a previously identified role for PAX3, that of regulator of an undifferentiated plastic state, may operate in melanocytes of normal skin. This role, possibly required for cellular response to environmental stimuli, may contribute to formation and development of melanocytic lesions in which PAX3 expression is prominent.

## Introduction

Cutaneous Malignant Melanoma is a highly aggressive form of skin cancer, thought to be derived from cutaneous melanocytes or melanoblasts. Its aggressiveness is attributed to frequent metastasis and high drug resistance. Intensive research into the mechanisms regulating melanoma tumourigenesis has included investigation of the factors and pathways of normal melanocyte development and function. One key factor that regulates melanocyte development from neural crest derived progenitor cells is the transcriptional factor PAX3.

PAX3 is very frequently expressed in melanomas and naevi [1]–[6]. In fact, PAX3 has been identified as a significant marker for melanoma staging [7], [8] and for detection of circulating melanoma cells [7]. It has also been identified as an immunogenic protein in melanomas [9]–[11], with several epitopes able to induce the host's immune response - stimulation of the immune response against *PAX3*-expressing tumour cells results in tumour growth suppression [9], [11].

PAX3 is a member of the PAX family of transcription factors which are highly conserved throughout phylogeny. All play a crucial role in embryogenesis but are also implicated in tumourigenesis [12]–[18]. Although PAX3 is recognised as a key embryonic regulator of melanocyte specification and development, its expression and function in differentiated epidermal melanocytes of adult human skin is largely unexplored and its role in melanoma remains unclear [19].

*Pax3* starts its expression in early embryos, in neural crest precursor cells that later give rise to melanocyte precursors, melanoblasts [19]–[24]. Here the main function of Pax3 besides neural crest specification is regulation of melanoblast survival and proliferation [23], [25]. Together with SRY (sex determining region Y)-box 10 (Sox10), Pax3 regulates expression of key melanocyte specification factor *Microphthalmia-associated transcription factor* (*Mitf*). It has been suggested that Pax3 controls a “nodal point” in melanocyte differentiation; it simultaneously activates *Mitf* and represses *Dopachrome-tautomerase (Dct)* transcription thus blocking terminal differentiation [26]. Once Mitf levels reach a certain threshold this repression is removed, allowing Mitf, in the presence of β-catenin, to activate *Dct* expression and melanocyte maturation. This model of regulation of melanocyte differentiation suggests that *Pax3* expression is either negligible or significantly reduced in adult differentiated melanocytes.

Several studies report *PAX3* expression in naevi and melanomas [2], [4], and show no expression in melanocytes of normal skin [5], [6], thus concluding that *PAX3* re-expression might be involved in melanocyte transformation. In contrast to this widely accepted assumption, other studies note *PAX3* expression in melanocytes of normal skin [22], [27], and more specifically its up-regulation as a result of the UV-induced loss of TGFβ signalling from keratinocytes [28]. Additionally, *PAX3* expression has been described in cultured melanocytes [3], [29] and melanoblasts with no significant change in levels of either mRNA or protein between these two cell types [29].

Here we confirm that PAX3 is present in the majority of naevus and melanoma cells. We also show conclusively that PAX3 is expressed in melanocytes of normal skin where it is co-expressed with known melanocyte markers. Since the precise role played by PAX3 in melanomas is not clear, we have looked into potential PAX3 regulated pathways within normal melanocytes. We have chosen pathway-representative markers that are also downstream targets of PAX3 and analysed their co-expression with PAX3 in normal skin melanocytes, naevi and melanomas. Interestingly, while the majority of PAX3-positive melanocytes in the epidermis of normal skin show a mature phenotype, a portion (around 20%) show a less differentiated and proliferative phenotype. Moreover, PAX3-positive melanocytes frequently show expression of BCL2-like 1 (BCL2L1, also known as Bcl-X, bcl-xL or BCL-XL/S), an antiapoptotic marker. Taken together these results indicate that melanocytes of normal skin display a phenotype that is predisposed to malignant transformation (antiapoptotic, undifferentiated and proliferative). We also show that PAX3- positive epidermal melanocytes of normal skin are molecularly distinct from those of the hair follicle, which like melanoma cells express Melanoma cell adhesion molecule (MCAM, also known as MUC18/CD146), associated with cell migration and melanoma progression and metastasis.

## Results

### PAX3 expression in normal skin, naevi and melanoma

**Figure 1:**
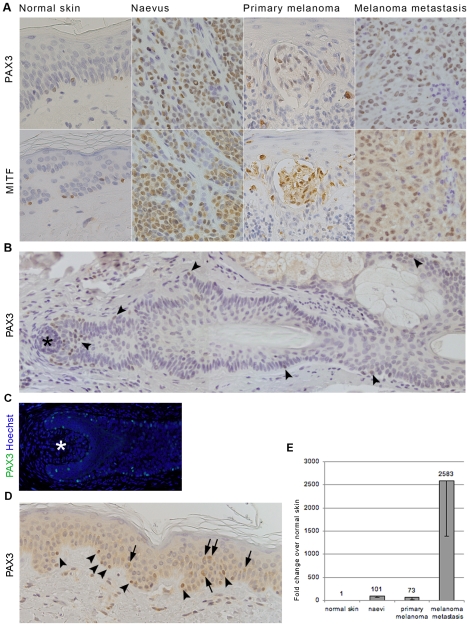
PAX3 expression in melanocytic and melanoma cells. A) Immunohistochemistry shows PAX3 expression (top panel) in representative samples of normal skin, naevus, primary melanoma and melanoma metastasis, compared to MITF expression in adjacent sections (bottom panel). B–D) Show the distribution of PAX3-positive melanocytes (arrowheads) in normal skin: along the hair follicle (early (B) and late (C) anagen); and in the epidermis (D). Arrows in (D) point to the cytoplasmic melanin deposit distinguishable from the nuclear PAX3 staining (arrowheads). Asterisk in (B) and (C) marks a dermal papilla. PAX3 was labelled with mouse monoclonal antibody (DSHB). E) *PAX3* expression was analysed by RT-PCR and the graph shows the mean fold increase of *PAX3* expression in naevi, primary melanomas and lymph node metastases normalised to the expression in normal skin.

**Figure 2:**
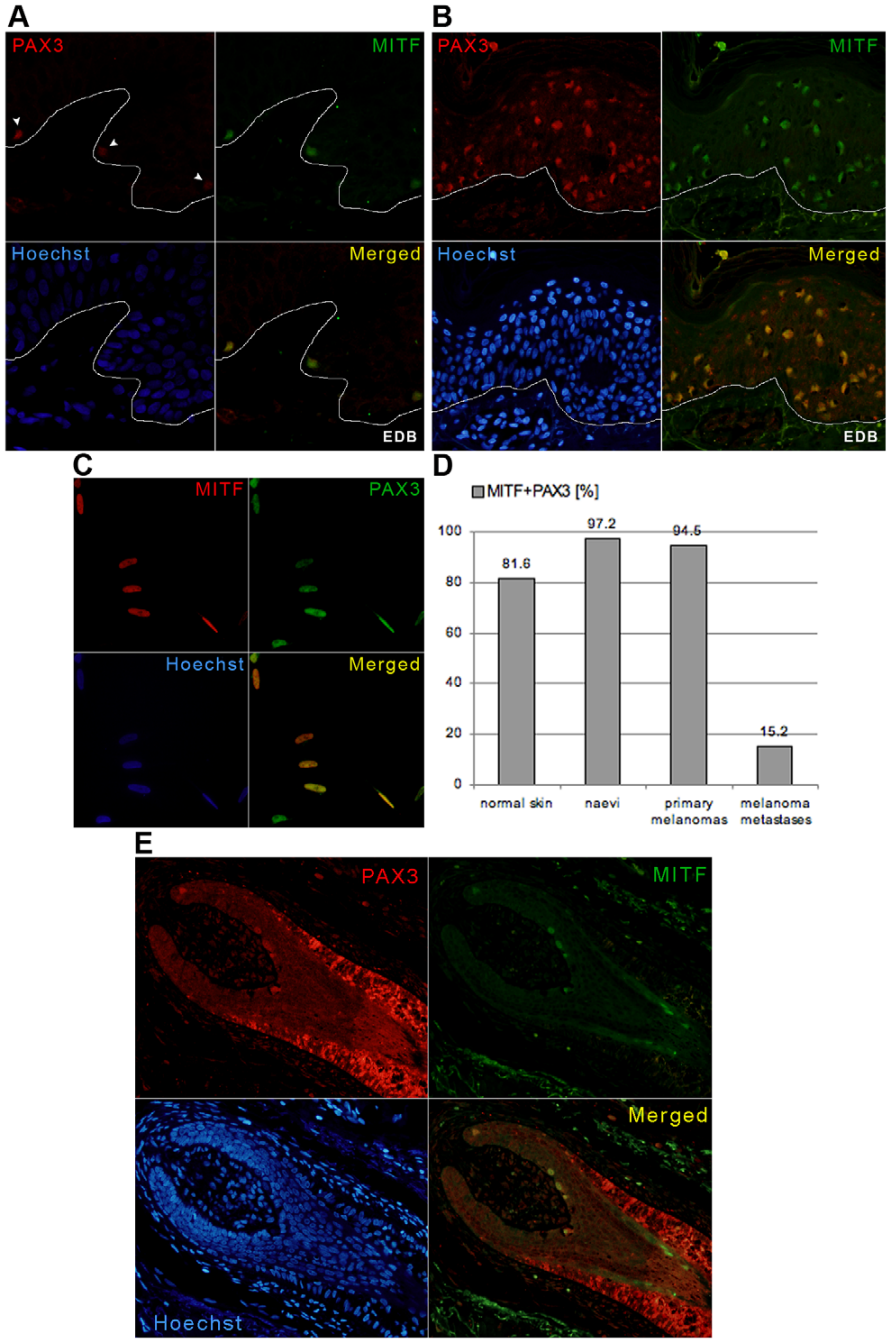
Co-expression of PAX3 and MITF in melanocytic and melanoma cells. A–C) Double immunofluorescent staining showing co-expression of PAX3 and MITF in: (A) the epidermal melanocytes of normal skin; (B) primary melanoma; and (C) in an A2058 metastatic melanoma cell line. Arrowheads in (A) show PAX3-positive normal epidermal melanocytes. Lines in (A) and (B) demarcate epidermal-dermal border (EDB). The variable PAX3 expression was clearly visible in the A2058 cell line (C). For all these experiments depicted in the figure, PAX3 was labelled with rabbit polyclonal antibody (Invitrogen). D) Graph showing the overall number of MITF and PAX3 double labelled cells in normal skins, naevi, primary melanomas and melanoma metastases. Each column represents a percentage of MITF-positive cells that are also PAX3-positive averaged across all samples. E) Double immunofluorescent staining shows PAX3 and MITF co-expressing melanocytes (yellow-orange) in the bulb of the hair follicle of normal skin. Note the single MITF-labelled melanocytes (green) at the base of the hair.

PAX3 expression was analysed by immunohistochemistry in tissue sections from paraffin embedded samples of normal skin, naevus, primary melanoma and melanoma metastases. Positive staining for PAX3 was obtained after performing heat-induced antigen retrieval in an EDTA/Tris buffer, pH 8.0 and incubating the primary antibody (anti-PAX3, DSHB) at room temperature (Figure 1A, top panel). Antigen retrieval using the most common 10mM citrate buffer, pH 6.0 with the same staining protocol, failed to produce a positive signal in several of these samples, and in those that were positive after citrate antigen retrieval, the intensity of staining was dramatically reduced. Negative controls included omitting the primary antibody from the procedure resulting in no immunohistochemical signal (not shown). Mouse monoclonal anti-PAX3 antibody (DSHB) has been previously confirmed by immunohistochemistry to be specific for the PAX3 protein [30]. PAX3-specific staining was also confirmed using a different commercially available anti-PAX3 antibody (Invitrogen) (Figure 2, A–C and E). However, we have observed less intensive staining overall with this polyclonal antibody (Invitrogen), compared to that observed with the monoclonal (DSHB).

All of the samples analysed showed positive PAX3 staining in more than 10% of cells (Figure 1A, top panel). In normal skin PAX3-positive cells showed a pattern of distribution that is characteristic of melanocytes; i.e. they were found at the epidermal-dermal boundary and along the hair follicle (Figure 1A–D). When adjacent sections were stained with MITF, a known marker of melanocytes, a comparable pattern of distribution was observed (Figure 1A, bottom panel).

The intensity of PAX3 staining varied between cells within the same lesion and between different samples. There was no obvious correlation between either intensity or number of PAX3-positive cells and lesion type. However, PAX3-positive cells of normal skin samples generally showed weaker staining and far fewer cells were positive, compared to those in naevi and primary melanoma samples. In contrast, melanoma metastases have an overall low level of PAX3 expression and these cells were mainly located at the periphery of the lesion.

Additional analysis by quantitative RT-PCR was performed on a separate set of samples, to confirm *PAX3* expression in normal skin, naevi, primary melanomas, and melanoma metastases. *PAX3* expression was detected in all naevi (5/5), primary melanomas (5/5) and melanoma metastases analysed (5/5), and in one out of two (1/2) normal skin samples. The highest levels of *PAX3* expression were found in melanoma metastases (mean fold change of 2583±1198), followed by naevi and primary melanomas with 101±39 and 73±55- fold change, respectively (Figure 1E). Even though these results suggest that *PAX3* might be up-regulated in advanced melanomas (melanoma metastases vs. primary melanomas) comparison with immunohistochemistry results show that expression levels are not due to increased amounts of *PAX3* per individual cell, but rather they reflect the number of cells expressing *PAX3* (Figure 1A). Nevertheless RT-PCR results confirm *PAX3* expression in melanocytes of normal skin.

### Characterisation of PAX3-positive cells: Co-expression of PAX3 and MITF

In order to validate PAX3 expression in naevi and melanomas as well as to confirm that PAX3-positive cells in normal skin are in fact melanocytes, co-staining of PAX3 and MITF was assessed by immunofluorescence. Indeed, in all of the samples analysed, including normal skin, PAX3-positive cells co-express MITF (Figure 2, A–C and E). However, in normal skin (as in other samples) a small number of cells showed only MITF and not PAX3 expression, highlighting the variation in normal melanocyte cell phenotype.

In normal skin samples 81.6% of all MITF-positive melanocytes were also PAX3-positive (Figure 2D). Both naevi and primary melanoma samples show similar numbers of MITF-positive cells that also express PAX3 (97.2% and 94.5%, respectively) (Figure 2D). Interestingly, melanoma metastases show significantly less PAX3-positive than MITF-positive cells (Figure 2D), and generally these were weakly stained for PAX3. However, this was observed primarily when using rabbit polyclonal antibody to PAX3 (Invitrogen) for immunofluorescent staining. When the mouse monoclonal anti-PAX3 antibody (DSHB) was used for immunohistochemistry staining, many more PAX3-positive cells were observed on the same sample. The discrepancy between these two results is probably due to the sensitivity of the two antibodies. Nevertheless, the intensity of PAX3 staining in individual cells within the same tissue sample varied when either antibody was used, whereas MITF levels were uniform across each sample (Figure 2, A–C). This is shown clearly in cells of the A2058 metastatic cell line (Figure 2C) and may reflect differential cell status/phenotype.

### PAX3 in melanocyte differentiation: Co-expression of PAX3 with MITF, HES1 and MLANA

**Figure 3:**
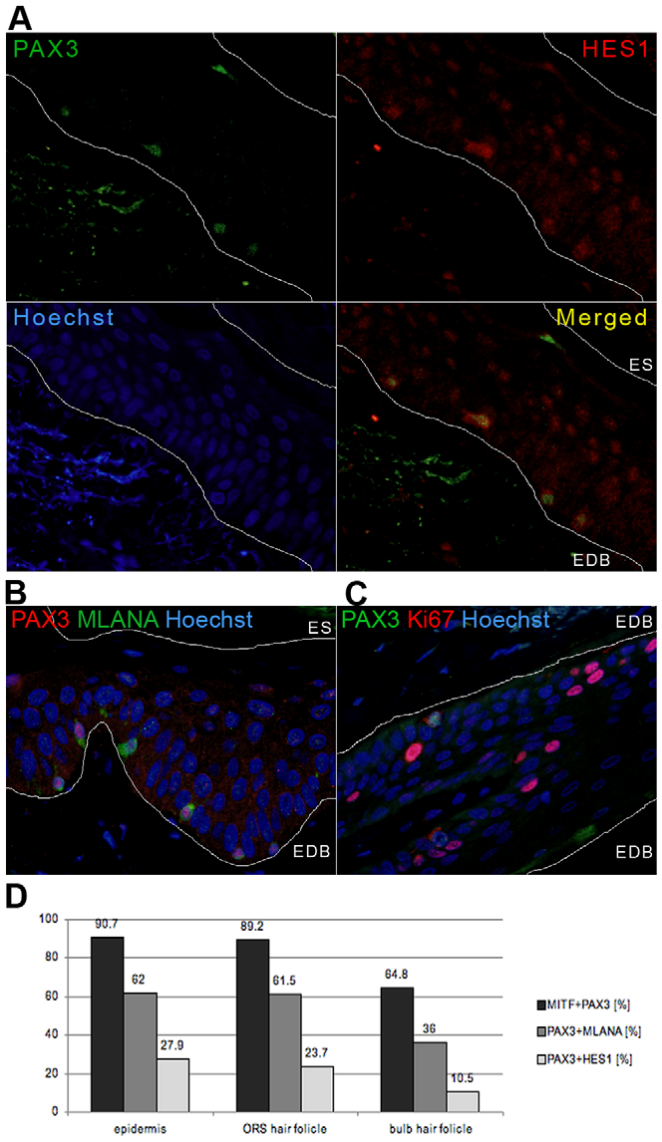
Co-expression analysis of PAX3-positive epidermal melanocytes of normal skin. Epidermal melanocytes of normal skin show a variable differentiation status: less differentiated melanocytes co-expressing PAX3 (mouse monoclonal antibody, DSHB) and HES1 (A); more differentiated co-expressing PAX3 (rabbit polyclonal, Invitrogen) and MLANA (B); and mature melanocytes expressing only MLANA (B). Single HES1-labelled cells in the epidermis are keratinocytes (A). C) PAX3 (mouse monoclonal antibody, DSHB) and Ki67 co-expressing melanocytes are also observed in the epidermis of sun-exposed skin. Lines in (A), (B) and (C) demarcate the epidermal-dermal border (EDB) or epidermal surface (ES). D) Graph shows the distribution of differentiation marker expression in normal skin melanocytes with respect to melanocyte location (in epidermis, outer root sheath (ORS), or hair follicle bulb).

Even though the overall percentage of MITF-positive melanocytes that were also PAX3-positive in normal skin was not significantly different in either benign or malignant melanocytic lesions (Figure 2D), the distribution of cells that co-expressed PAX3 and MITF differed between the epidermis and hair follicles of normal skin. Around 90% of MITF-positive melanocytes in the epidermis and the outer root sheath (ORS) of the hair follicle were PAX3-positive, compared to only 65% of those in the bulbar area (Figure 3D). Interestingly there was a distinct location in the hair bulb, at the very base of the hair, populated exclusively by MITF-positive melanocytes (Figure 2E).

**Figure 4:**
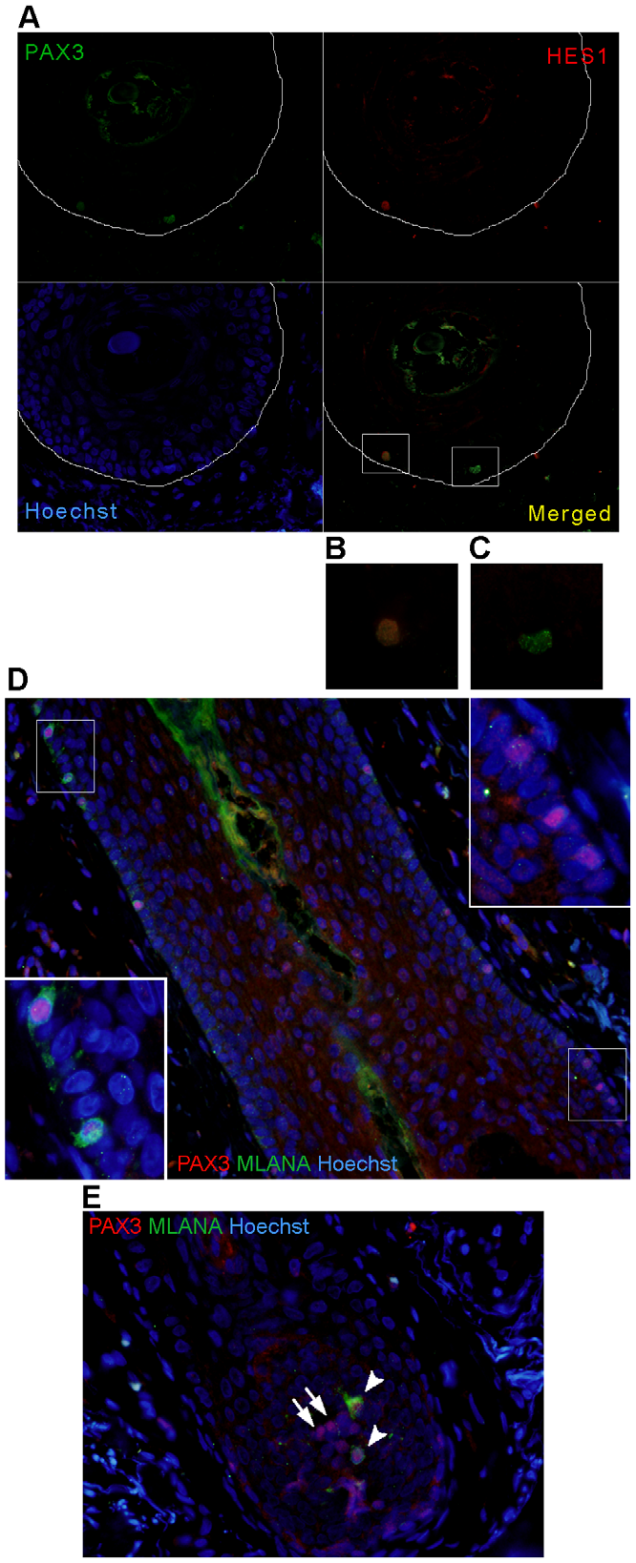
Co-expression analysis of PAX3-positive follicular melanocytes. A) The transverse section of the hair follicle shows both PAX3 and HES1 co-expressing (enlarged in B), and single PAX3-expressing (enlarged in C), melanocytes in the outer root sheath (ORS). The line circumscribes the hair follicle. PAX3 was labelled with mouse monoclonal antibody (DSHB). D) The longitudinal section of the hair follicle shows PAX3 and MLANA co-expressing (enlarged in the insert on the left) and single PAX3-expressing (enlarged in the insert on the right) melanocytes in the outer root sheath (ORS). E) Single PAX3-expresing (arrows) and PAX3 and MLANA co-expressing (arrowheads) melanocytes in the matrix of the hair bulb. PAX3 was labelled with rabbit polyclonal antibody (Invitrogen).

Given this difference between epidermal and follicular melanocytes, we thought it necessary to further characterise PAX3-positive melanocytes in the normal skin. We therefore co-stained PAX3 cells with Hairy and enhancer of split 1 (HES1) (Figure 3A and 4A–C) or Melan-A (MLANA, also known as MART-1) (Figure 3B and 4D, E), markers of a less or more differentiated cell phenotype respectively. Upon analysis of the distribution of double-labelled cells we found that HES1 was expressed in a proportion of PAX3-positive melanocytes, while cells that were only HES1-positive were identified as keratinocytes [31], [32] (Figure 3A). The number of PAX3-positive melanocytes co-expressing HES1 was lowest in the matrix of the hair bulb (10.5%), compared to the ORS (23.7%) and the epidermis (27.9%) (Figure 3D). By contrast, MLANA was expressed in more that 60% of both epidermal and ORS PAX3-positive melanocytes, compared to 36% of those in the matrix of the hair bulb (Figure 3D). We have also observed a significant number of single MLANA-labelled melanocytes (Figure 3B) (30.5% in the epidermis, 3.9% in ORS, and 40% in the hair bulb).

This indicates that melanocytes, both follicular and epidermal, have a variable differentiation status: from less differentiated PAX3, MITF and HES1-positive to more differentiated PAX3, MITF and MLANA-positive. The observed PAX3-negative, MLANA-positive epidermal melanocytes may represent mature terminally differentiated melanocytes.

In addition, Yang and colleagues [28] have recently proposed that in UV-treated skin TGF-β signalling from keratinocytes is downregulated, which increases PAX3 expression in epidermal melanocytes and stimulates their proliferation. In order to check if PAX3-positive melanocytes observed here are proliferating we co-stained PAX3 with a marker of proliferation, Ki67, and found 18.1% of epidermal melanocytes in samples of sun-exposed skin (scalp) to be double-labelled (Figure 3C). In contrast to this, in samples that had relatively little sun exposure (breast) one single double labelled cell was observed in the epidermis (accounting for 1.4% of PAX3-positive cells).

### PAX3 in cell survival: Co-expression with BCL2L1

**Figure 5:**
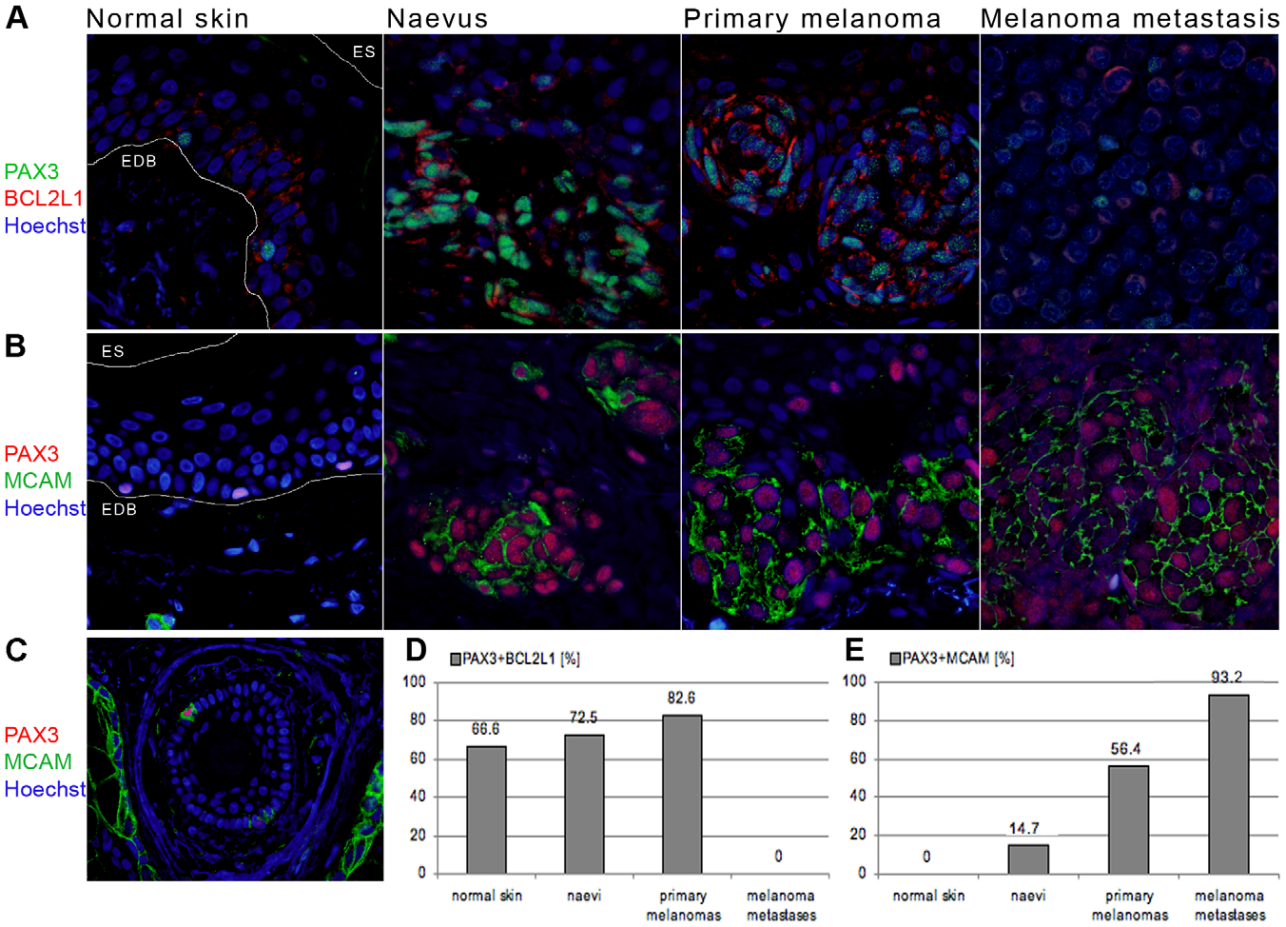
Co-expression of PAX3 with markers of cell survival and migration in melanocytic and melanoma cells. A) Double immunofluorescent staining showing PAX3 (mouse monoclonal antibody, DSHB) and BCL2L1 co-expression in representative samples of normal skin, naevus, primary melanoma and melanoma metastasis. B) PAX3 (mouse monoclonal antibody, DSHB) and MCAM co-expression in normal skin (epidermal melanocytes), naevus, primary melanoma and melanoma metastasis. Lines in (A) and (B) demarcate the epidermal-dermal border (EDB) or epidermal surface (ES). C) In contrast to the epidermal melanocytes, some PAX3-positive melanocytes in the outer root sheath (ORS) co-express MCAM. D) Graph showing the overall number of PAX3, BCL2L1 double-labelled cells in normal skins, naevi, primary melanomas and melanoma metastases. Each column represents a percentage of PAX3-positive cells that are also BCL2L1-positive, averaged across all samples. E) Graph showing the overall number of PAX3, MCAM double-labelled cells in normal skins, naevi, primary melanomas and melanoma metastases. Each column represents a percentage of PAX3-positive cells that are also MCAM-positive, averaged across all samples.

To further characterise the phenotype of PAX3-positive melanocytes relative to melanoma cells, expression of an antiapoptotic factor BCL2L1 was assessed in PAX3-positive cells (Figure 5A). We have observed that a similar proportion of PAX3-positive cells were also BCL2L1-positive in melanocytes of normal skin, in naevi and in melanoma cells in all of the samples analysed (Figure 5D). The exception is melanoma metastases, in which BCL2L1 was detected in only one sample (out of four) and both PAX3 and BCL2L1 were weakly stained and very rarely co-stain. BCL2L1 expression in epidermal keratinocytes, as reported previously [33], served as an internal positive control for the staining.

BCL2L1 expression in normal PAX3-positive melanocytes indicates that they might utilise the same cell survival regulatory mechanism as melanoma cells, in order to sustain continuous renewal in the ever-changing environment of the epidermis. It is possible that one of the roles PAX3 plays in adult melanocytes (similar to that in development) is to ensure their maintenance and survival, while regulating their specification and proliferation.

### PAX3 in cell migration: Co-expression with MCAM

Expression of the melanoma progression marker MCAM was also analysed relative to PAX3. MCAM expression was observed in most naevi and melanoma samples analysed; 80% (4/5) naevi samples, 66.7% (4/6) primary melanomas and 100% (5/5) of melanoma metastases. There was no MCAM expression in epidermal melanocytes of normal skin (Figure 5B). Notably we frequently observed MCAM expression in hair follicle cells of normal skin [34]–[36], mainly in the ORS and not exclusively in melanocytic cells (Figure 5C). MCAM expression in the endothelial cells of the blood vessels served as an internal positive control [36], [37].

In naevi samples MCAM expression was observed in specific areas, mainly in nests of cells in the dermal component of the naevus and only a small proportion (14.7%) of PAX3-positive cells were MCAM-positive (Figure 5B, E). Compared to naevi, primary melanomas showed MCAM expression in a larger proportion of PAX3-positive cells, 56.4% (Figure 5B, E), which are again primarily located in the dermal component of the lesion. Samples of melanoma metastases showed MCAM expression in the majority (93.2%) of PAX3-positive cells (Figure 5B, E).

In general, MCAM co-expressed with PAX3 in all MCAM-positive samples. In accordance with previous reports [34], the number of MCAM expressing PAX3-positive cells increased with malignant and particularly metastatic transformation. The expression of MCAM in follicular melanocytes, in contrast to the absence of expression in epidermal melanocytes, highlights again the difference between these two melanocyte populations.

## Discussion

### PAX3 expression in normal skin, naevi and melanoma

In agreement with previous reports, we confirm here that PAX3 is expressed in all melanoma and naevi samples analysed. There is however a paucity of studies analysing PAX3 expression in differentiated melanocytes in human skin. Traditionally considered a critical embryonic regulator, PAX3 has been suggested to maintain melanocyte stem cells in the bulge area of the hair follicles in mice [26], but was thought to be down-regulated upon terminal differentiation of melanocytes. This belief has been supported by reports detailing the absence of PAX3 expression in melanocytes of normal human skin in contrast to naevi and melanomas [5], [6]. Our results show conclusively that PAX3 is expressed in both epidermal and follicular melanocytes of human skin. Furthermore, co-expression with MITF confirms the melanocytic origin of these PAX3-positive cells. We also show here, for the first time, the variable phenotype of epidermal melanocytes of normal skin.

The observed frequency of PAX3 expression in naevi, melanomas and specifically in melanocytes in normal skin is much higher than reported elsewhere [5], [6]. One possible explanation could be the methodology we used, namely EDTA/Tris antigen retrieval buffer, rather than the citrate buffer commonly used by other researchers, which was, in our experience, less efficient. It might also reflect the influence of environmental factors, namely sun exposure and solar radiation. It has recently been suggested that in UV-treated skin TGF-β signalling from keratinocytes is down-regulated, increasing PAX3 expression in epidermal melanocytes thus stimulating their proliferation [28]. All the samples used in this study are collected from people living in Australia, where the solar radiation levels are in the high to extreme range for much of the year. A recent report from New Zealand [27] shows PAX3 expression in all naevi, melanomas and normal skin samples, confirming our findings. Further studies are required to investigate differences in PAX3 expression in skin samples derived from the Southern vs. the Northern hemispheres.

Normal skin samples used in this study, mainly originating from anatomic locations frequently exposed to the sun (such as limbs and face), were free of any melanocytic lesion having been used for other diagnostic purposes. We have included here two normal skin samples from breast reductions which would have lower sun exposure levels and these samples also show PAX3-positive melanocytes in the epidermis. We have looked to see if PAX3-positive melanocytes in our samples were proliferative, by co-staining with Ki67. In our samples of sun-exposed skin, 18.1% of epidermal PAX3-positive melanocytes were proliferative, but the majority were not. This was in contrast to samples subjected to low levels of sun exposure, where we observed one single PAX3, Ki67-positive cell. However, to accurately address the question whether the high frequency of PAX3 expression in normal melanocytes observed in this study, relative to previously published papers, is caused in any way by the extent of sun exposure, a more thorough analysis of skin subjected to chronic and/or acute UV exposure relative to sun-protected skin, would need to be performed.

### PAX3 expression in melanocytes of normal adult skin: A role in regulating differentiation

PAX3 and MITF are crucial regulators of melanocyte development and not surprisingly have overlapping expression. To some extent they lie on opposing sides of the differentiation pathway, PAX3 being upstream of MITF and MITF initiating the melanogenic cascade and differentiation. We were interested to see if there is an association between expression of PAX3 and MITF (indicated by their co-expression) and the stage of melanocyte differentiation (based on the cell location, mainly in the hair follicle), but found no correlation. Studies on mice and human skin both show the existence of skin stem cells in the lower permanent portion of the hair follicle, or bulge [35], [38], [39]. It is reported that mouse bulge melanocyte stem cells are PAX3-positive and MITF-negative [40]; however no reports confirm this to be the case in human skin. In this study we were not able to find such cells. Instead the majority of melanocytes we observed, both in the epidermis and the outer root sheath of the hair follicle, were PAX3 and MITF-positive. In contrast to this, the matrix of the hair bulb has far fewer PAX3 and MITF-positive melanocytes and the base of the hair comprises solely MITF-positive (PAX3-negative) melanocytes. Similarly, the number of PAX3, MLANA-double-labelled melanocytes in the epidermis and ORS were around 60%, but were much lower (around 36%) in the hair matrix. This would indicate that some epidermal melanocytes resemble more the undifferentiated transient amplifying cells of the growing hair rather than differentiated cells of the hair matrix.

On the other side of differentiation is the transcription factor HES1, suggested to prevent premature differentiation of melanocytes. Hes1 is reported to be a mediator of Notch signalling in skin development and in hair follicle maintenance where it regulates commitment of epidermal keratinocytes to terminal differentiation [32], [41]. Hes1 is expressed in spinous layers and mediates spinous cell gene induction and thus terminal differentiation of epidermal keratinocytes [32]. In a mature hair follicle it is most prominent in the inner root sheath, in matrix cells committed to terminally differentiate to form the hair shaft [31], [32]. On the other hand, in the melanocytic lineage, Notch signalling, acting through Hes1, plays a crucial role in survival of immature melanoblasts and melanocyte stem cells, by preventing initiation of apoptosis [42]. Moreover, it prevents differentiation of melanoblasts and melanocyte stem cells before they reach the hair bulb and regulates their proper location in the ORS and in the hair matrix [43]. Therefore it is not surprising that we see a decrease in the number of PAX3 and HES1-positive cells in the hair bulb compared to the ORS (10.5% and 23.7%, respectively). What is surprising is that around 28% of PAX3-positive epidermal melanocytes are also HES1-positive, indicating they are not terminally differentiated.

In summary, it seems that epidermal melanocytes are not all terminally differentiated and similar to those in the hair follicle, they exhibit variable differentiation status with persistent PAX3 expression. Melanocytes in the ORS of the hair follicle are mostly PAX3 and MITF-positive and not all are fully differentiated (23.7% HES1-positive, 61.5% MLANA-positive). In the matrix of the hair bulb the number of PAX3-positive melanocytes is lower (around 65%), with only a small proportion still showing a less differentiated phenotype (10.5% HES1-positive and 36% MLANA-positive). Epidermal PAX3-positive melanocytes show a similar profile to those in the ORS (27.9% HES1-positive, 62% MLANA-positive). Interestingly, there are a similar number of MLANA-positive (but PAX3-negative) melanocytes in the epidermis (30.5%) and the hair matrix (40%), in contrast to the ORS where such cells are rare (3.9%). PAX3 expression seems to correlate with undifferentiated (co-expressing HES1 and MITF) and differentiating (co-expressing MLANA and MITF) cells, whereas it might be diminished in terminally differentiated cells (PAX3-negative but MLANA or MITF-positive) that are further along the continuum of differentiation. It is possible that PAX3 is involved in maintenance of melanocyte “stemness”, at least while they are migrating towards their destination, either in the hair follicle or the epidermis.

### PAX3 in apoptosis

It is also very likely that PAX3 is involved in keeping melanocytes alive, since three quarters of epidermal and more than half ORS and matrix PAX3-positive melanocytes show expression of the PAX3 target, antiapoptotic BCL2L1. PAX3 is previously reported as an antiapoptotic regulator in tumours, including melanoma [44]–[49]. Several known antiapoptotic factors, such as tumour suppressors p53, PTEN and BCL2L1, are involved in Pax3-induced cell survival [48], [50], [51]. *BCL2L1* is directly transcriptionally regulated by PAX3, and in rhabdomyosarcoma treatment with *PAX3* or *BCL2L1* antisense oligonucleotides, individually or in combination, decreases cell viability to a similar extent, suggesting that they lie in the same antiapoptotic pathway [51]. Similarly, BCL2L1 is associated with melanoma cell survival, since its expression correlates with melanoma progression [52], [53] and treatment with antisense oligonucleotides resulted in reduction of cell viability [54], [55]. However, its expression is observed in naevi and normal skin [52], [53] and in melanocytes it also affects cell viability [55].

As expected we have observed positive BCL2L2 staining in all normal skin, naevi and primary melanoma samples analysed. The majority of PAX3-positive cells in melanomas, naevi and normal skin co-express BCL2L2 at a similar frequency. These results support the potential role of PAX3 as one of many cell survival regulators. The results also indicate that normal melanocytes might utilise the same cell survival regulatory pathway as melanoma cells, both in the epidermis and in hair follicles.

### PAX3 in migration

The cell adhesion molecule MCAM has been associated with melanoma progression and metastatic potential [56]–[59]. Even though MCAM is frequently expressed in naevi [36] its increased expression in melanomas shows significant correlation with poor disease free survival and mortality [36], [56]–[58], [60], [61]. Up-regulation of MCAM, together with loss of keratinocyte-dependence, is one of the crucial events that allows melanoma cells to invade the dermis and progress to vertical growth phase (for review see [62]). MCAM can mediate both homotypic adhesion between melanoma cells, promoting local tumour growth, and heterotypic adhesion between melanoma cells and endothelial cells of blood vessels, facilitating metastatic spread [59], [63], [64].

Pax3-transfected melanocyte cells show increased expression of MCAM, both at an mRNA and protein level, suggesting that MCAM is a downstream target of Pax3 [65], [66]. Our results confirm co-expression of PAX3 and MCAM in melanomas and naevi, mainly in the cells that form nests located in the dermal component of the lesion, with melanoma samples showing larger numbers of PAX3, MCAM-positive cells compared to naevi. This indicates that some PAX3-positive cells in naevi and melanomas have the capability to undergo vertical spread/migration. It suggests that regulation of migration might be one of the roles of PAX3 in melanoma, as it is in development, where Pax3 regulates cMet [67].

The frequent observation of MCAM expression in cells of the hair follicle might be associated with downwards growth into the dermis and migration of follicular cells. The observation that some PAX3-positive melanocytes in the ORS show MCAM expression, in contrast to those in the epidermis, suggests that MCAM is associated with migration of normal melanocytes from the bulge area to the developing hair follicle and to the epidermis. Upon reaching the epidermis, MCAM expression is downregulated. Alternatively, epidermal melanocytes do not arise and migrate from the bulge, but rather arise from interfollicular melanocyte stem cells, similar to those believed to maintain interfollicular epidermal homeostasis [68]–[70]. Even though some epidermal melanocytes resemble the transient amplifying melanocytic cells of the hair follicle with respect to their differentiation status, they are not thought to have the same migratory propensity.

In summary, in addition to detection in the majority of naevus and melanoma cells, we conclusively show PAX3 expression in melanocytes of normal skin. Epidermal PAX3-positive melanocytes, like follicular cells, exhibit a variable differentiation status; the majority show a more mature phenotype, but a proportion (around 20%) show a less differentiated and proliferative phenotype. Moreover, these melanocytes frequently show expression of a cell survival marker. Our results suggest that a previously identified role for PAX3, as a regulator of an undifferentiated plastic state, may operate in melanocytes of normal skin. This role, possibly required for cellular response to environmental stimuli, may predispose a proportion of these cells to a successful malignant transformation as they are antiapoptotic, undifferentiated and proliferative.

In addition, our findings that PAX3 expression is specific for both the melanocytic lineage and melanocytic lesions, could have potential applications in the clinical setting, as an immunohistochemical assay for the differential diagnosis of melanoma. The effectiveness of the commercially available anti-PAX3 antibodies, and optimisation of staining protocols shown in this paper, might also prove useful in a clinical setting.

## Materials and Methods

### Sample collection

De-identified archival tissue samples were obtained from several pathology laboratories in accordance with the Human Research Ethics Committee of Edith Cowan University (ethics approval code 07-189 MEDIC). Written consent was obtained for the use of patient archival tissue samples. Samples were previously diagnosed by certified pathologists. 4µm thick formalin fixed paraffin embedded tissue sections were used for immunohistochemistry and immunofluorescence. The following samples were analysed by immunohistochemistry and immunofluorescence: 10 normal skins (including 3 hairy, 2 limb and 2 breast skin samples, and 3 samples had no anatomic location specified), 14 naevi (including 5 intradermal, 6 compound and 3 junctional samples), 15 primary (including 8 superficial spreading melanoma samples, 2 of radial growth phase, and 1 sample each of acral lentiginous, nodular, neurotropic, recurrent and lentigo maligna melanoma) and 5 systemic melanoma metastases (2 small bowel and 3 lymph node). Tissue samples characterized here as normal skins were free of any melanocytic lesion, having been used for other diagnostic purposes (solar keratosis, seborrhoeic keratosis and granulomatous folliculitis) or tissue obtained after breast reduction. Normal skin samples originated mainly from limbs or face (referred to as sun-exposed skin) or breast (referred to as reduced sun-exposed skin). Additionally, a separate set of 12 archival samples, including 2 normal skin, 5 naevi and 5 primary melanomas as well as 5 cryopreserved lymph node melanoma metastases were analysed by qRT-PCR.

### RNA extraction and qRT-PCT

Total RNA was extracted from the paraffin embedded and cryopreserved tissue samples using Aurum Total RNA Mini Kit (Bio-Rad) according to the manufacturer's recommendations. Slight modifications were made to the protocol used for paraffin embedded samples. Mainly, sections were dewaxed in Xylene and absolute Ethanol (each twice for 10 minutes), before tissue was scratched from the slides and lysed in a mixture of Tissue Lysis Buffer (High Pure RNA Paraffin Kit, Roche), Proteinase K and 10% SDS by incubation at 55°C overnight.

The quality and integrity of extracted RNA was assessed by gel electrophoresis and subsequent amplification of the housekeeping gene *GAPDH*. 250ng of the total RNA was reverse transcribed with Omniscript RT kit (Qiagen), according to the manufacturers' instructions and PCR was performed using Taq DNA Polymerase (Qiagen). Primers used were, for *GAPDH*: 5′-GGG TGT GAA CCA TGA GAA GT-3′ (forward) and 5′-GAC TGT GGT CAT GAG TCC T-3′ (reverse) (Takeuchi et al. 2003); and for *PAX3*: 5′-AGT GAG ATT ACG CGC TAG -3′ (forward) and 5′-CCA GCG GAA GAC CAG AAA C-3′ (reverse). Real time PCR was performed using iQ SYBR Green Supermix (Bio-Rad). Each sample was run in duplicate and assays included negative controls (reagents without RNA or cDNA) and positive controls (plasmid DNA containing a *PAX3* insert). The calibration curve was generated using the threshold cycle (Ct) of 8 serial dilutions of plasmid DNA template with known copy number. The Ct value of the sample was interpolated from the standard curve and mRNA copy number, mean value and standard deviation were calculated using iQ5 RealTime Detection System Software (Bio-Rad Laboratories). The increase in the mean values of mRNA copy number for each sample relative to that in the normal skin was expressed as a fold-change increase in mRNA.

### Antibodies

The following primary antibodies were used: mouse monoclonal to PAX3 (DSHB, 1/10), rabbit polyclonal to PAX3 (Invitrogen, 1/500), mouse monoclonal to MITF (Merck, 1/20), mouse monoclonal to MLANA (Merck, 1/50), rabbit polyclonal to HES1 (Abcam, 1/100), rabbit monoclonal to BCL2L1 (Abcam, 1/50), rabbit monoclonal to MCAM (Abcam, 1/500) and rabbit polyclonal to Ki67 (Abcam, 1/25). For immunofluorescent staining the following secondary and tertiary antibodies were used: anti-mouse conjugated AlexaFluor-488 (1/500), anti-rabbit conjugated AlexaFluor-488 (1/500), anti-mouse conjugated AlexaFluor-546 (1/500), or biotinylated anti-rabbit IgG (1/500) linked to streptavidin-conjugated AlexaFluor-546 (1/500). All antibodies were diluted in PBST (0.2% TritonX-100 in PBS) together with 1%NGS for immunofluorescent staining.

### Immunohistochemistry

Paraffin embedded sections were dewaxed in xylene (3×5 minutes) and dehydrated in ethanol series (3×5 minutes in 100% ethanol, 1 minute in each of 95% and 70% ethanol, and 3×10 dips in ddH_2_0). Antigen retrieval was routinely performed in EDTA/Tris, pH 8.0, by heating for 3×5 minutes in a microwave oven, at approximately 750 Watts. Slides were left to cool to room temperature for at least 20 minutes. Sections were washed in PBS and endogenous peroxidases were blocked with 3% H_2_0_2_ for 10 minutes. After rinsing in PBS, sections were blocked with 10% FCS for one hour, followed by incubation with primary antibodies for one hour at room temperature. After washing in PBS, the secondary antibody linked to biotin (Dako, LSAB kit) was applied and sections were incubated for 20 minutes at room temperature. After washing in PBS-Tween20 (0.05%), sections were incubated with streptavidin conjugated horseradish peroxidase (Dako, LSAB kit) for 20 minutes at room temperature, washed in PBS-Tween20, then incubated with DAB (Dako) for several minutes. The reaction was stopped by washing in ddH_2_0, and the signal was enhanced by applying a solution of CuSO_4_ and NaCl, for 3 minutes. Hematoxylin staining was performed before the slides were mounted with DPX neutral mounting medium. Results were analysed on an Olympus BX51 microscope and images were captured using an Olympus DP71 camera. Controls with primary antibodies withheld were immunonegative. The specific staining of nuclear markers PAX3 and MITF was generally distinguishable from the cytoplasmic melanin deposit in the tissue by their sub-cellular localisation. Intensity of PAX3 staining was compared across all samples analysed and described as either weak or strong.

### Immunofluorescence

The same dewaxing, rehydration and antigen retrieval procedures described above were followed for immunofluorescence. Sections were blocked with 10%NGS for one hour at room temperature, followed by incubation with primary antibodies at 4°C overnight (for rabbit PAX3 and MITF co-staining) or at room temperature for one hour (for all other antibodies). After washing in PBS, sections were incubated with the appropriate secondary antibody (followed by tertiary only for rabbit PAX3 and HES1) for one hour and washed in PBS. For counterstaining Hoechst 33342 was used. Sections were mounted with FluorSave Reagent (Calbiochem) and analysed on an epifluorescent Olympus BX51 microscope equipped with an Olympus DP71 camera. Controls with primary antibodies withheld were immunonegative. For each sample, quantification of cells positive for a given marker was performed by analysis of 5–10 representative regions of the section. The number of double-labelled cells was calculated relative to the number of cells positive for a comparative marker and expressed as a percentage.
